# Defect Engineering: Can it Mitigate Strong Coulomb Effect of Mg^2+^ in Cathode Materials for Rechargeable Magnesium Batteries?

**DOI:** 10.1007/s40820-024-01495-1

**Published:** 2024-09-20

**Authors:** Zhengqing Fan, Ruimin Li, Xin Zhang, Wanyu Zhao, Zhenghui Pan, Xiaowei Yang

**Affiliations:** 1 School of Chemistry and Chemical Engineering, Shanghai Jiao Tong University, Shanghai, 200240 People’s Republic of China; 2 School of Chemistry and Chemical Engineering, Taiyuan University of Technology, Taiyuan, 030024 People’s Republic of China; 3 School of Materials Science and Engineering, Tongji University, Shanghai, 201804 People’s Republic of China

**Keywords:** Rechargeable magnesium battery, Sluggish diffusion kinetic, Defect engineering, Cathode materials, Ion migration

## Abstract

Rechargeable magnesium batteries (RMBs) have been considered a promising “post lithium-ion battery” system to meet the rapidly increasing demand of the emerging electric vehicle and grid energy storage market. However, the sluggish diffusion kinetics of bivalent Mg^2+^ in the host material, related to the strong Coulomb effect between Mg^2+^ and host anion lattices, hinders their further development toward practical applications. Defect engineering, regarded as an effective strategy to break through the slow migration puzzle, has been validated in various cathode materials for RMBs. In this review, we first thoroughly understand the intrinsic mechanism of Mg^2+^ diffusion in cathode materials, from which the key factors affecting ion diffusion are further presented. Then, the positive effects of purposely introduced defects, including vacancy and doping, and the corresponding strategies for introducing various defects are discussed. The applications of defect engineering in cathode materials for RMBs with advanced electrochemical properties are also summarized. Finally, the existing challenges and future perspectives of defect engineering in cathode materials for the overall high-performance RMBs are described.

## Highlights

- The underlying migration mechanism of Mg^2+^ in cathode materials and roles of defects in Mg^2+^ migration in cathode materials were studied.
- Applications of defect engineering to Mg^2+^ migration in cathode materials and the strategies for introducing various defects were summarized.
- New development directions of defect engineering in cathode materials for rechargeable magnesium battery were prospected

## Graphical abstract

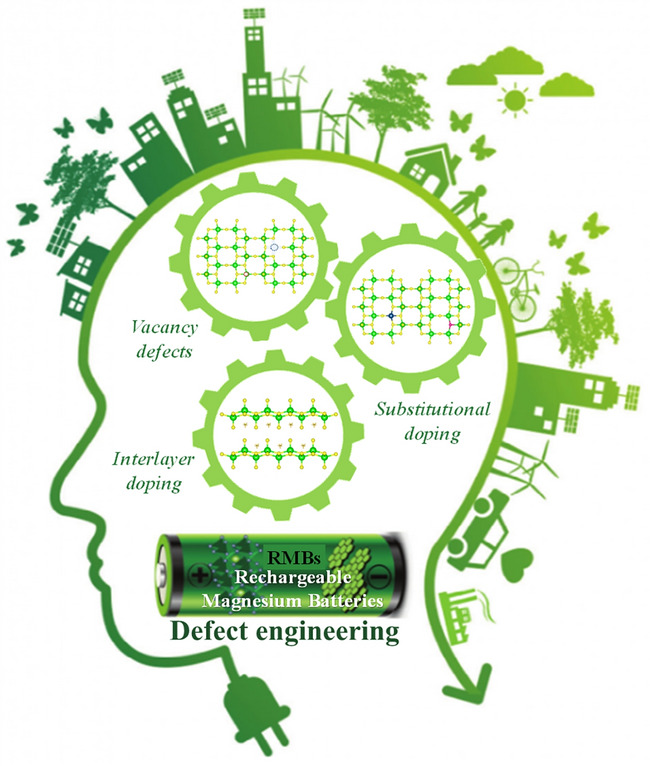

## Introduction

**Fig. 1:**
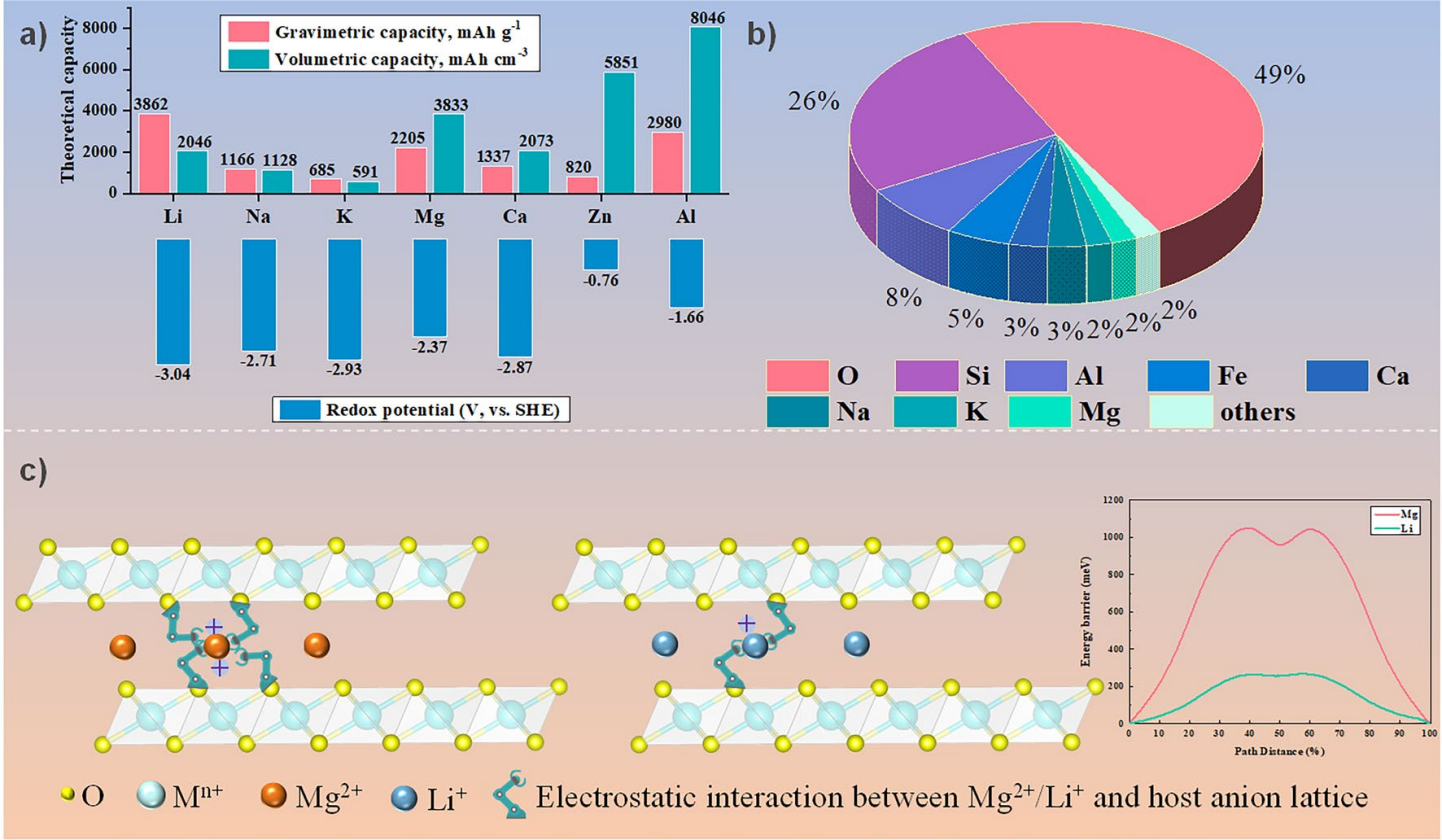
**a** Capacities and redox potentials of different metal anodes, **b** elemental abundance in the Earth’s crust, **c** a vivid illustration of the comparison of the reaction kinetics of Mg^2+^ and Li^+^ in layered cathode. The inset shows Mg^2+^ exhibits sluggish diffusion kinetics in NiO_2_ compared to Li^+^

Considering energy crisis and environmental pollution are the two major concerns of today’s society, it is urgent to develop sustainable energy storage from intermittent solar and wind sources to replace traditional fossil fuels [1–4]. To date, lithium-ion batteries (LIBs) have been widely dominated in our daily lives ranging over portable electronics, electric vehicles, and smart grids [5–8]. Nevertheless, issues with safety, cost, and resources have hampered the utilization of LIBs in large-scale energy storage systems [9–11]. Therefore, developing the “post LIB” system to meet the rapidly increasing demand has become one of the most important scientific and societal challenges. Among various battery systems, rechargeable magnesium batteries (RMBs) have been considered as a promising candidate due to the apparent metrics of Mg metal anode including Earth’s crust (~ 2%), less prone to dendrite deposition, and high volumetric capacity (3833 mAh cm^−3^ for Mg). (Fig. 1a, b) [12–18].

Although great progress has been achieved recently, RMBs still have a long way to their maturity due to the limitations of the irreversibility of Mg anodes, low-efficiency Mg electrolytes, and lacking high-performance Mg^2+^ host cathode materials [19–29]. Compared to anode and electrolyte, the cathode material lies at the heart of the RMB system to determine the overall energy and power densities. However, the development of RMB cathode materials still faces scientific challenges related to the strong electrostatic interaction between Mg^2+^ and host anion lattices, which is caused by the inherently strong ionic polarization force of bivalent Mg^2+^ [30–34]. Therefore, most cathode materials (e.g*.*, Chevrel phase, spinel, layered, and olivine structures) show sluggish diffusion kinetics of Mg^2+^, further leading to serious voltage polarization/hysteresis and a low magnesiation degree [35]. Moreover, although the ionic radii of Mg^2+^ and Li^+^ are comparable (0.72 Å for Mg^2+^ and 0.76 Å for Li^+^), Mg^2+^ has a greater polarizing power (3.85 e Å^−2^ for Mg^2+^ and 1.73 e Å^−2^ for Li^+^) [36]. Thus, some classical materials can reversibly intercalate Li^+^ but exhibit poor electrochemical activity against Mg^2+^ (Fig. 1c) [37–41].

**Fig. 2:**
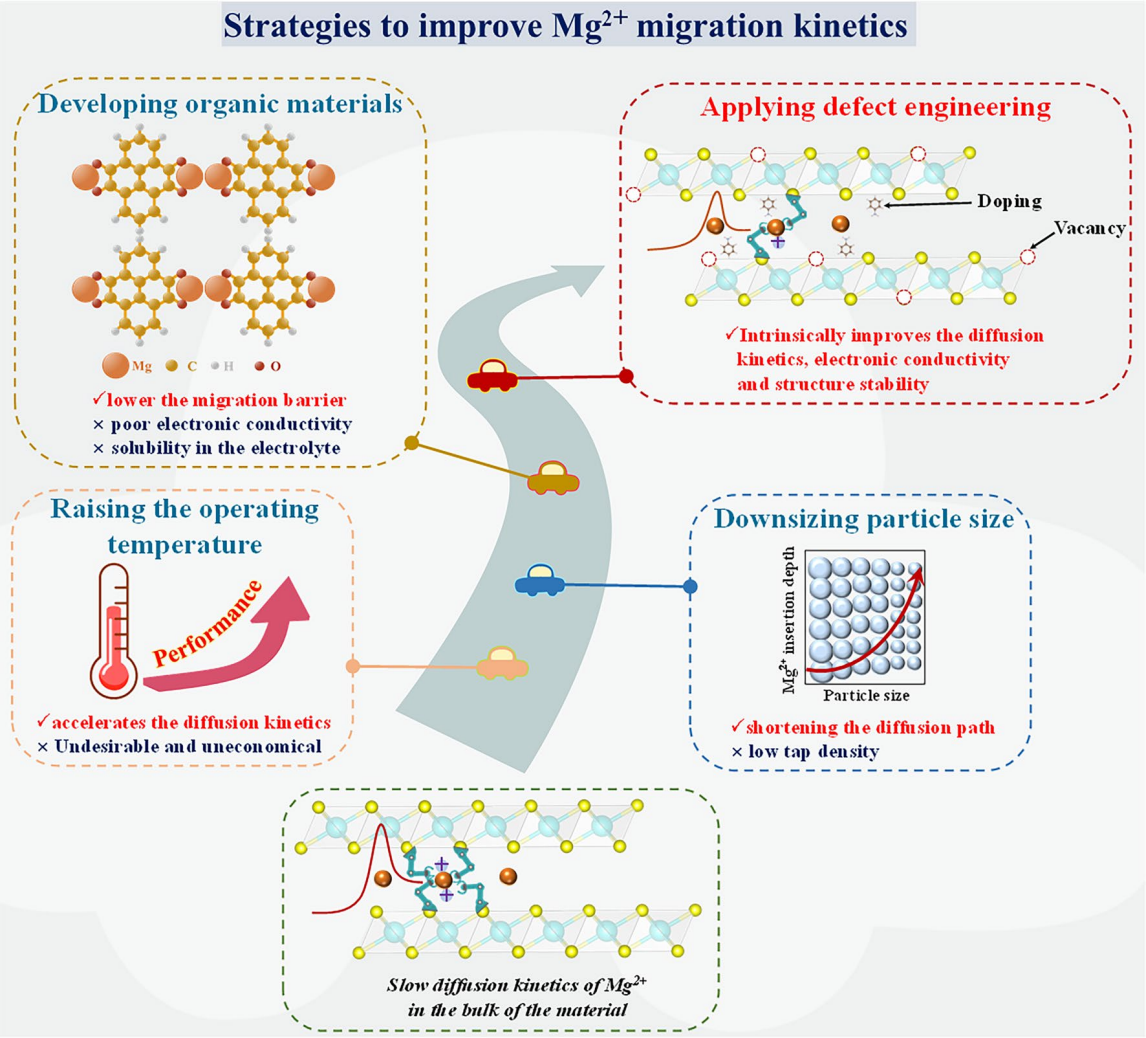
Strategies to improve Mg^2+^ migration kinetics and reversibility in cathode materials

Various strategies, such as tailoring material size, elevating the operating temperature, and designing organic materials (Fig. 2), have been recently developed to improve Mg^2+^ storage and reversibility in cathode materials [42]. These tactics can facilitate the reaction dynamics to some extent, but will also lead to certain detriments. For example, the nanosized particle can improve the diffusion of Mg^2+^ by shortening the diffusion path in the cathode material accompanied by reducing the energy density of RMB due to the low tap density of the ultrasmall particles [30]. Furthermore, raising the operating temperature of the battery certainly accelerates the diffusion kinetics of Mg^2+^, while it is not a desirable and economical manner for the practical application of RMBs [43]. Regarding the organic cathode materials, although the weaker intermolecular interactions and larger intermolecular spacing in the organic compounds will lower the Mg^2+^ migration barrier and provide more Mg storage sites [32, 44–46], they usually suffer from poor electronic conductivity and high solubility in the electrolyte, which result in a low capacity and unsatisfactory cycling stability [47–49]. Therefore, it is essential to develop an effective strategy that can effectively improve the Mg^2+^ diffusion kinetics while minimizing the collateral adverse effects.

Defect engineering is an effective strategy to improve ion diffusion dynamics, electronic conductivity, and structural stability of electrode materials, which has been successfully demonstrated in various battery systems [9, 50–52]. However, a comprehensive and detailed review of the fundamentals, research advances, and future challenges of defect engineering in cathode materials for RMBs has not been published so far. This review first highlights the fundamental understanding of the intrinsic mechanism of Mg^2+^ migration and the corresponding affecting factors. Then, we briefly discuss the positive effects of intentionally introduced defects in the cathode materials and various strategies for introducing defects. Moreover, the applications of defect engineering in cathode materials for advanced RMBs are systematically summarized. Finally, we describe the existing challenges and perspectives about the future direction of defect engineering in cathode materials for high-performance RMBs.

## Understanding of the Underlying Migration Mechanism of Mg^2+^ in Cathode Materials

As we all know, the cation (such as Li^+^ and Mg^2+^) migration determines the design science of cathode materials for rechargeable batteries. The diffusion energy barriers of ions are destined to be different for different diffusion paths. Therefore, understanding the diffusion of ions in the bulk phase of a material is essential to improve the ion diffusion kinetics. For example, intercalation-type inorganic materials are the only commercialized cathode materials for LIB because they deliver relatively long cycle stability and high energy density. Therefore, they have also been extensively studied in RMBs, which are mainly classified into Chevrel phase (CP), spinel, layered, and olivine cathodes. However, due to the slow diffusion kinetics of Mg^2+^ in the solid state, inorganic materials with high potential, high capacity, and stable cyclability for RMBs have not been reported so far. To solve this challenge, it is first necessary to understand the underlying migration mechanism of Mg^2+^ in the cathode materials, where the migration trajectories/pathways of Mg^2+^ and the factors affecting Mg^2+^ migration are the two keys. Thus, in this section, the migration pathways of Mg^2+^ in cathode materials with different structures/compositions and the corresponding factors affecting the migration of Mg^2+^ will be discussed systematically.

### Migration Pathways of Mg^2+^

**Fig. 3:**
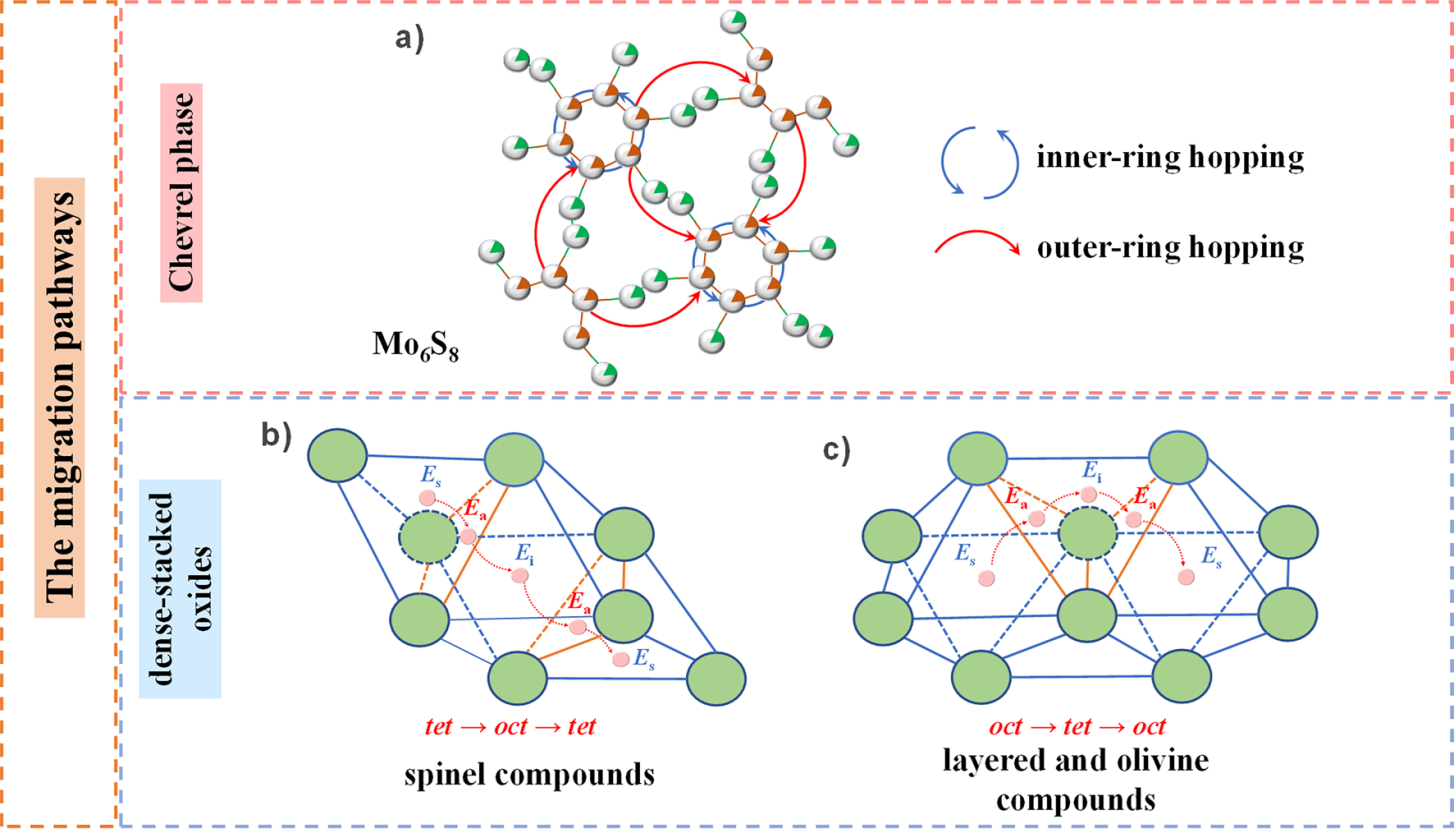
The migration pathways of Mg^2+^ in **a** CP Mo_6_S_8_, **b**, **c** several typical dense-stacked oxide materials

Generally, the migrating routes for Mg^2+^ in different structural materials are diverse due to different compositions and geometrical configurations. Thus, based on previous works, we will briefly discuss the migration pathways of Mg^2+^ in different cathode materials with a classical structure. In one of the pioneer works, Aurbach et al*.* reported that the CP Mo_6_S_8_ can be successfully used as cathode materials for RMBs because the Mo_6_S_8_ shows a unique character where six Mo atoms bind to form an octahedron interconnected by eight S anions [53]. The structure of CP possesses a quasi-simple-cubic packing of Mo_6_S_8_ superanions, which has three highly symmetric positions: 3*b* (the center of the Mo_6_S_8_ superanion), 3*a* (the body center of the superanion cubic), 9*d* (the face center of the superanion cubic which located in the middle of two 3*a* sites) [53]. Among the three types of sites, only the three-dimensional (3D) channels formed by 3*a* and 9*d* can accept the insertion of Mg^2+^ [54]. The insertion positions of Mg^2+^ around 3*a* form a six-membered ring called the inner sites, and the positions near 9*d* are deemed the outer sites [55]. In addition, there are two hopping modes for Mg^2+^ insertion into CP Mo_6_S_8_: the hopping within the six-membered ring (inner-ring hopping) and the hopping between neighboring inner and outer sites (outer-ring hopping), respectively (Fig. 3a) [56]. Moreover, the calculations indicate that the migration of Mg^2+^ in the inner sites has a small barrier at dilute Mg concentration [53] and the hop from the inner sites to the outer sites requires overcoming a large energy barrier [13]. Therefore, at a low Mg^2+^ concentration, only the 3*a* site is filled and the extra Mg^2+^ undergoes an outer-ring hopping between the neighboring inner sites, inserting into the external site [53].

Spinel, layered, and olivine structures are also three typical types of cathode materials for RMBs. The spinel (e.g., Mn_2_O_4_) and layered compounds (e.g*.*, NiO_2_) are face-centered cubic (*fcc*) structures, while the olivine compounds (e.g*.*, FePO_4_) show hexagonal close-packed (*hcp*) structures. In dense-stacked oxygen (or sulfur, selenium, tellurium, etc.) structures, tetrahedral (*tet*) and octahedral (*oct*) interstitial sites share a single face (Fig. 3b, c). Within these structures, direct diffusion between equivalent sites is unlikely to occur (either *tet* → *tet* or *oct* → *oct*) because Mg^2+^ needs to cross through a narrow O–O bond, which requires an ultrahigh energy barrier to be overcome [50]. Therefore, Mg^2+^ usually migrates through the shared face between tetrahedra and octahedra, *i.e.*, *tet* → *oct* → *tet* (Fig. 3b) or *oct* → *tet* → *oct* (Fig. 3c). The diffusion route of Mg^2+^ in spinel Mn_2_O_4_ structure is typically *tet* → *oct* → *tet*. The Mg^2+^ initially resides in the stable *tet* site (with energy *E*_s_), then travels through the three-coordinated anionic face (with energy *E*_a_) shared with the adjacent intermediate *oct* site to reach the *oct* site (with energy *E*_i_), and finally follows a symmetric pathway to the next equivalent stable site (*i.e.*, the *tet* site) [57]. In olivine FePO_4_ and layered NiO_2_ structures, the migration of Mg^2+^ follows a similar pattern, but initially stays at the stable *oct* site, then diffuses through the intermediate *tet* sites, and finally reaches the next equivalent stable site.

### Factors Affecting the Migration of Mg^2+^

**Fig. 4:**
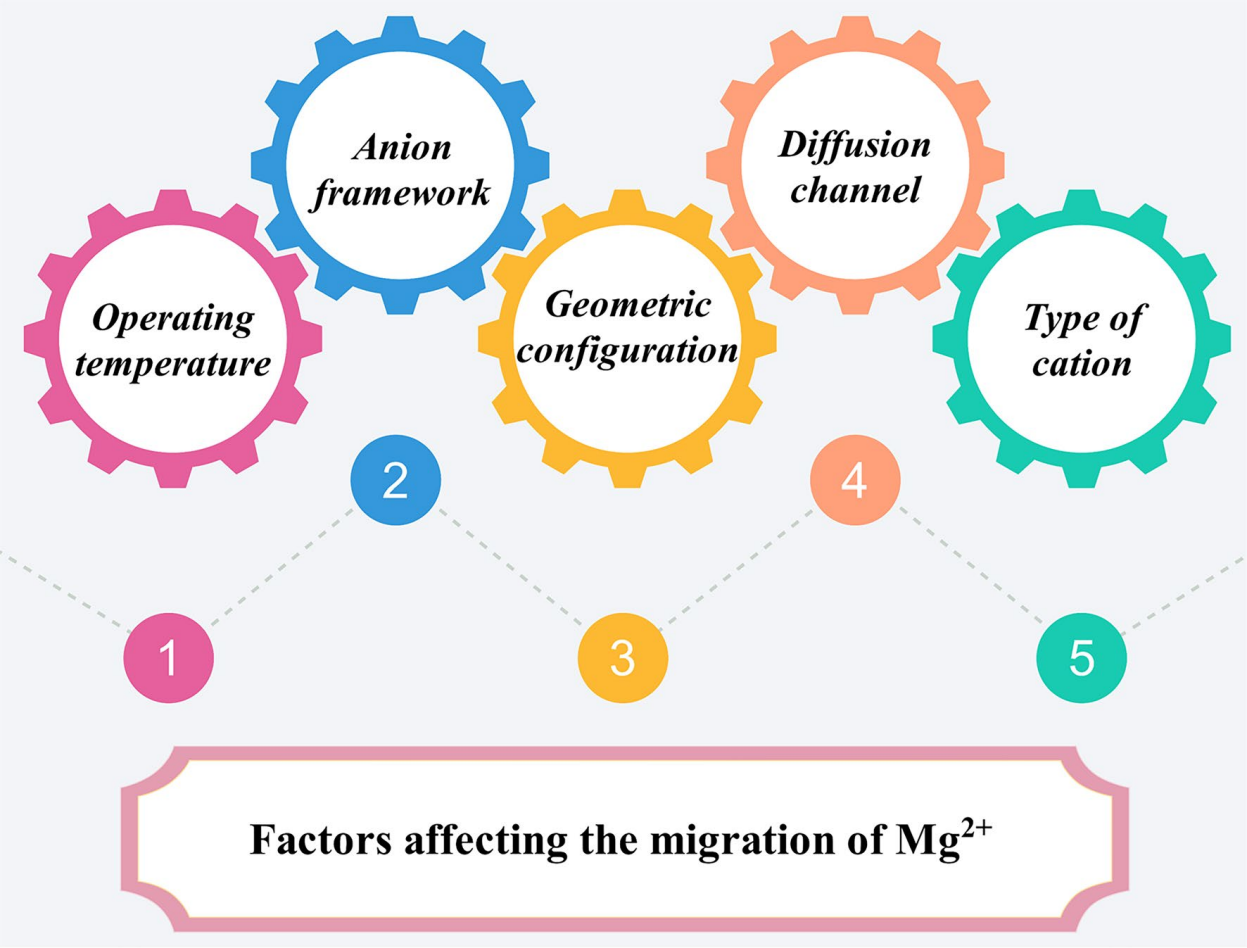
The main factors affecting the migration of Mg^2+^

In general, the migration kinetics of Mg^2+^ will directly affect the electrochemical performance of the RMBs. In addition to the charge density of the Mg^2+^ itself, the chemical composition of the cathode material and the structural characteristics will affect the ion diffusion. For ideal intercalated materials where the migration barrier, Δ*E*, for each ion hop is not dependent on the local degree of ion ordering/disordering, the ion diffusion coefficient (*D*) can be shown as [58, 59]:

1 $$D = \rho \lambda^{2} \varGamma$$

where *ρ* is a geometric factor that represents the dimensionality and connectivity of the interstitial network determined by the symmetry of the sublattice at the interstitial site, and is equal to $z/2d$ in which *z* is the coordination number and *d* is the lattice dimension. Typically, crystal structures with high coordination number *z* show large geometric factors. The *λ* is the hop distance between neighboring interstitial sites. *Γ* is the hop frequency, which can be expressed as [58]:

2 $$\varGamma = \nu^{*}{\text{exp}} \left( { - \frac{\Delta E}{{kT}}} \right)$$

where v^*^ is a vibrational factor, $\Delta E$ is the diffusion energy barrier, *k* is the Boltzmann constant, *T* is temperature. From Eqs. (1) and (2), it is known that the ion diffusion coefficient is mainly correlated with the operating temperature, diffusion energy barrier, geometric configuration of the host material, diffusion channel of intercalated ions, and type of cation. Combined with the analysis in Sect. 2.1, it is known that the Mg^2+^ migration pathways in the cathode material are primarily related to the anion framework. Therefore, the diffusion energy barrier is primarily influenced by the anion framework. In conclusion, the factors affecting the Mg^2+^ diffusion are mainly operating temperature, anion framework, geometric configuration of the host material, diffusion channel of Mg^2+^, and type of cation in the host material (Fig. 4). These points will be discussed in detail as following.

#### Operating Temperature

**Fig. 5:**
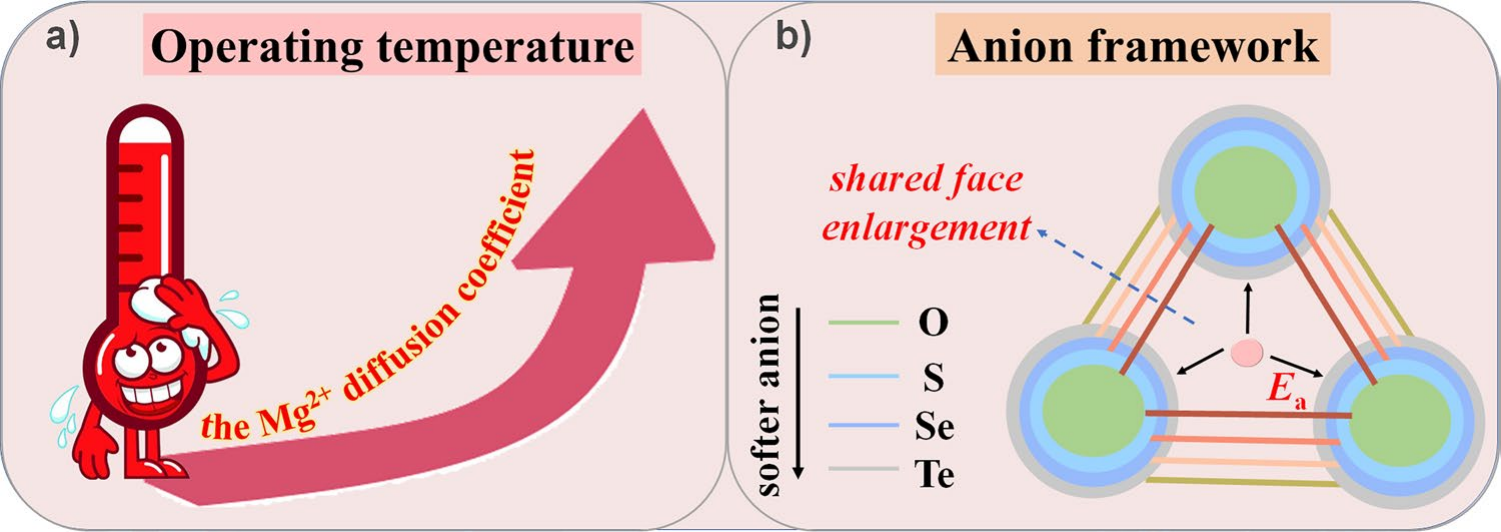
Effect of **a** operating temperature and **b** anion framework on the Mg^2+^ diffusion

First of all, the operating temperature will undoubtedly affect the diffusion of Mg^2+^. Elevating the temperature will promote the migration of Mg^2+^ in the host material lattice (Fig. 5a). Therefore, increasing the temperature will also increase the Mg storage performance of cathode materials. It should be noted that it is not advantageous to raise the temperature all the time. The increase in temperature will also accelerate the occurrence of side reactions, causing capacity decay, and overly high temperatures will also cause safety concerns. Moreover, elevating the operating temperature requires a suitable electrolyte, which means that the electrolyte needs to be able to maintain electrochemical stability and a suitable electrochemical window at higher temperatures. Finally, the higher operating temperature may not be applicable for the use of the battery in daily life. Nevertheless, increasing the operating temperature facilitates the prescreening of the cathode for RMBs [42].

#### Anion Framework

Secondly, the diffusion energy barrier is also a vital factor affecting the migration of Mg^2+^. The magnitude of the diffusion energy barrier is mainly dominated by the shared surface between the insertion sites, which is composed of anions. Therefore, the anion framework of the host material affects Mg^2+^ migration. On the one hand, compared to oxides, “softer” anion frameworks with smaller electronegativity (such as sulfides, selenides, or tellurides) have weaker interactions with Mg^2+^, favoring a lower migration barrier of Mg^2+^ [60–63]. On the other hand, the radius of the anion also affects the Mg^2+^ migration. The larger anion radius, the larger shared surface between the insertion sites, thus providing a broader diffusion channel (Fig. 5b) [64]. However, it should be noted that the softer anion frameworks (sulfides/selenides) typically exhibit low voltage plateaus, leading to a lower energy density.

#### Geometric Configuration

**Fig. 6:**
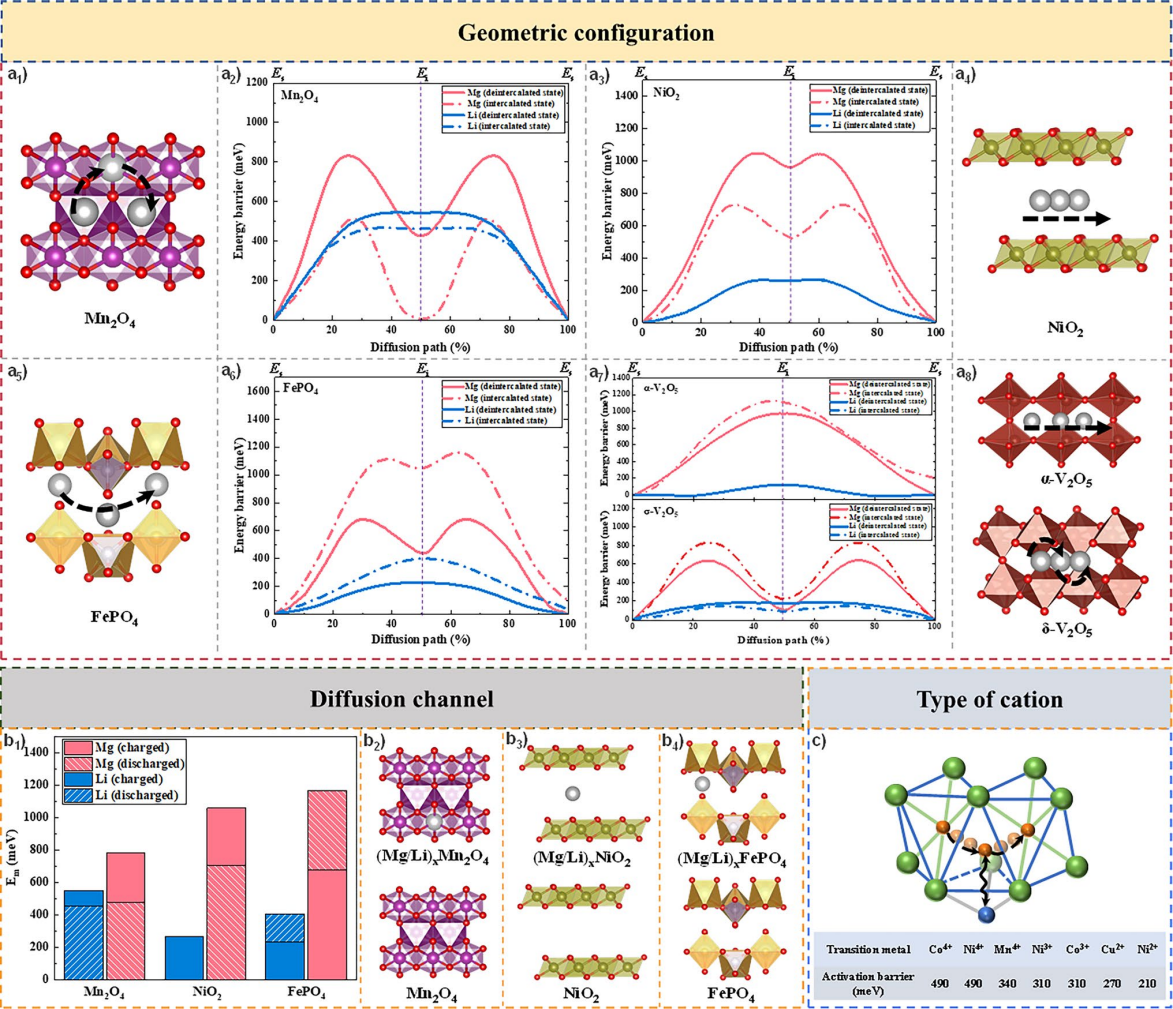
**a**_**1**_–**a**_**6**_ Calculated migration energies *E*_m_ for different intercalating ions in the spinel Mn_2_O_4_, layered NiO_2_ structures, and olivine FePO_4_, in the deintercalated/charged limit (solid lines), and the intercalated/discharged limit (dashed lines) [57]. **a**_**7**_–**a**_**8**_ Calculated migration energies *E*_m_ for different intercalating ions in *α*-V_2_O_5_ and *δ*-V_2_O_5_ in the empty lattice (solid) and dilute vacancy (dashed) concentration limits [65]. **b**_**1**_–**b**_**4**_ A summary of the migration barriers *E*_m_ for different intercalating ions in the different states [57]. **c** Calculated energy barrier at the intermediate site with strong repulsion from different transition metal cations [66]

Thirdly, the geometric configuration affects the migration of Mg^2+^ in the host material, taking layered NiO_2_, olivine FePO_4_, spinel Mn_2_O_4_, and *δ*-V_2_O_5_ as examples for comparison. Moreover, as mentioned above, the coordination number affects the geometry factor and thus the diffusion coefficient. The anion coordination preferences of several different cations are as follows: Li^+^ is most often found in four-coordination (4) and Mg^2+^ in six-coordination (6) [50, 57]. In layered NiO_2_ and olivine FePO_4_, the stable insertion site for Mg^2+^ is the *oct* site, which is also preferred coordination (six-coordination) and requires migration through the intermediate *tet* site. Because a large energy barrier needs to be overcome when migrating through sites with lower coordination, Mg^2+^ has a higher migration barrier (*E*_m_) compared to Li^+^ in both layered NiO_2_ and olivine FePO_4_ (Fig. 6a_3_, a_4_ and a_5_, a_6_) [57]. In spinel Mn_2_O_4_, the stable and intermediate insertion site for Mg^2+^ is the *tet* site and *oct* site, respectively. Therefore, the absolute value of the site energy difference $\left| {E_{i} - E_{s} } \right|$ for Mg^2+^ diffusion is smaller in Mn_2_O_4_ (Fig. 6a_1_, a_2_) than that of NiO_2_ (Fig. 6a_3_, a_4_). In *δ*-V_2_O_5_, the stable insertion site of Mg^2+^ is in the corner-shared tetrahedral position, which can be considered a nominal “4 + 2” coordination. The diffusion path of Mg^2+^ is “4 + 2” → “square pyramid” → “4 + 2” [57]. Since the coordination variation between the stable and intermediate sites is smaller (“4 + 2” → 5 → “4 + 2” compared to 4 → 6 → 4), the absolute value of the site energy difference $\left| {E_{i} - E_{s} } \right|$ is expected to be smaller (Fig. 6a_7_, a_8_) [65].

#### Diffusion Channel

Fourthly, the diffusion channel of intercalated ions also affects diffusion. For spinel Mn_2_O_4_ and layered NiO_2_, in the discharged state, the migration energy barrier of Mg^2+^ in the host material is lower than that of charged state, whereas for olivine FePO_4_, the migration energy barrier of Mg^2+^ is higher in the discharged state than that in the charged state (Fig. 6a_1_–a_6_ and 6b_1_–b_4_). Such a huge difference originates from the disparate migration paths of Mg^2+^ with 3D in spinel structures, two-dimensional (2D) in layered structures, and one-dimensional (1D) in olivine structures. In the spinel structure, the increased concentration of inserted Mg^2+^ facilitates the reduction of the electrostatic interaction with nearby transition metals, which further stabilizes the intermediate *oct* sites. In the layered structure, the increased concentration of inserted ions favors the expanding layer spacing, which facilitates the insertion of subsequent ions. However, in olivine structures, an increase in the concentration of the pre-inserted ion elevates the migration energy barrier of the subsequent ion, or even blocks the migration channel. It can be seen that keeping the diffusion channel unblocked is essential for the diffusion of Mg^2+^.

#### Type of Cation

Except for the above factors, the valence state (ionic radius) of the transition metal cations in the host materials also affects the migration of Mg^2+^. Similar to anions, electrostatic interactions also exist between the cations in the host material and the inserted ions. The more charge the host cations carry, the greater the electrostatic repulsion between them and the inserted Mg^2+^. Consequently, the higher the valence of the host cation, the higher the migration energy barrier of the Mg^2+^ (Fig. 6c) [66]. Additionally, cations may migrate to occupy Mg^2+^ insertion sites during the charge/discharge process, which may also affect the subsequent diffusion behavior of Mg^2+^.

## Roles of Defects in Mg^2+^ Migration in Cathode Materials

**Fig. 7:**
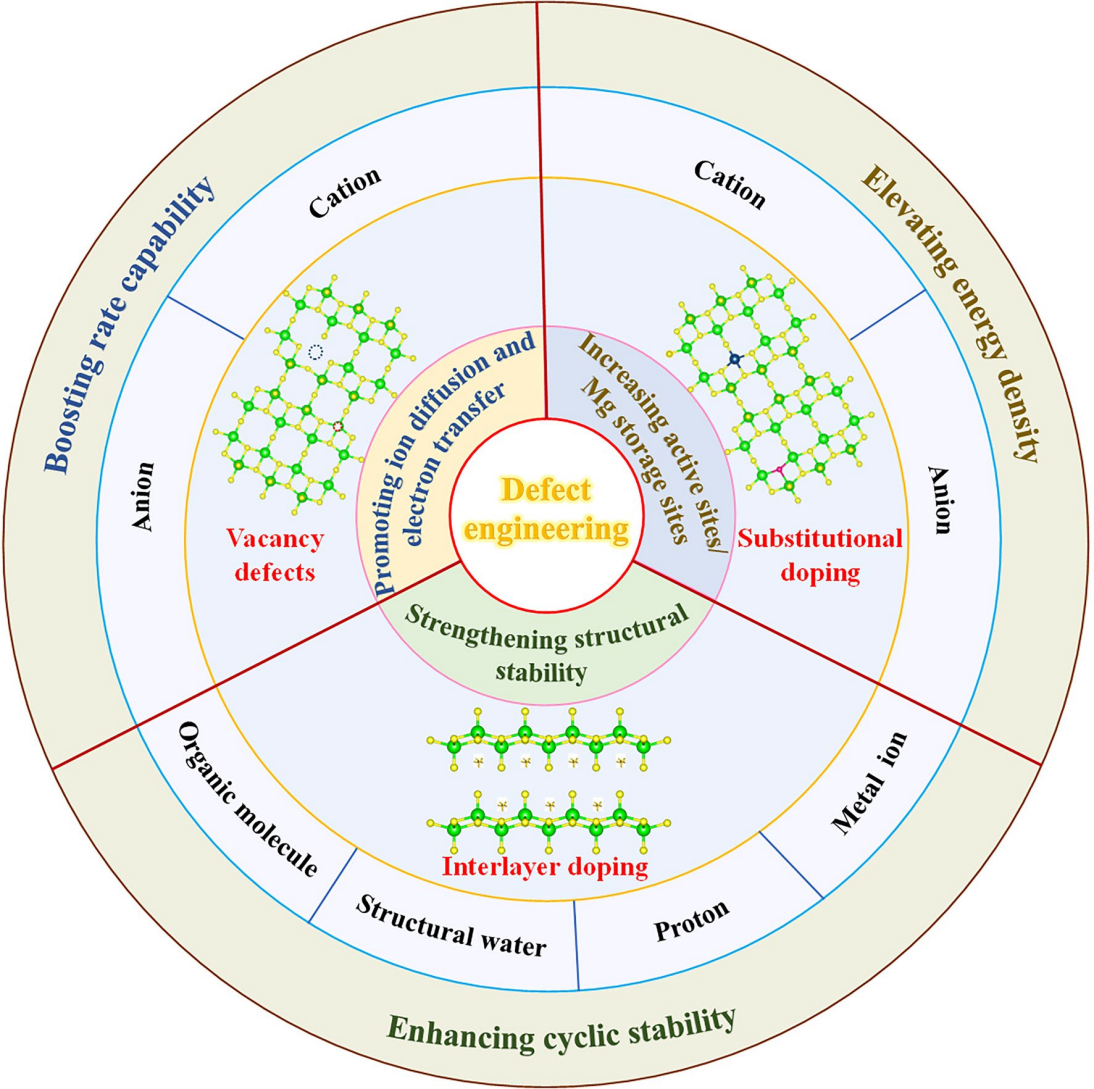
Overview of defect engineering in cathode materials for RMBs

Recently, defect engineering in cathode materials has been successfully demonstrated to boost the slow diffusion kinetics of Mg^2+^, thus improving the electrochemical performance of RMBs. As shown in Fig. 7, the introduction of defects can effectively modify the electrostatic interaction of Mg^2+^ with the anionic and cationic frameworks of the host material, reducing migration energy barrier, and raising electronic conductivity. Moreover, defect engineering can not only serve to increase the active sites or Mg storage sites in the host material, increasing the Mg storage capacity of cathode material but also contribute to enhancing the structural stability of the host material. This section details the effect of introducing defects on the cathode material.

### Accelerating Mg^2+^ Diffusion and Electron Transfer

Sluggish diffusion kinetics is a painful concern for inorganic cathode materials for RMBs. As mentioned previously, the migration barrier of Mg^2+^ in the same inorganic material is much higher due to carrying more charge, although the ionic radius is almost the same as that of Li^+^. The strong electrostatic interaction of Mg^2+^ with anions and cations in the host material is one of the most important causations. By introducing defects, the diffusion of Mg^2+^ can be effectively enhanced. The introduction of vacancies, either anionic or cationic, can weaken the electrostatic interactions between Mg^2+^ and the host framework, thus reducing the barrier. In particular, introducing cationic vacancies can also keep the diffusion channel unblocked, avoiding the generation of deleterious phases clogging the diffusion of Mg^2+^. Simultaneously, doping by extra elements or original elements (*i.e.*, self-doping) could create vacancies, thus improving the migration of Mg^2+^. Likewise, the introduction of vacancies or doping can change the original electronic arrangement of the host material and the valence state of the original element, or trigger shallow impurity levels, thereby reducing the bandgap and heightening the electronic conductivity of the host material [9, 51, 67, 68]. For example, Jin et al. prepared oxygen vacancy-rich 2D black TiO_2-*x*_ (B–TiO_2-*x*_) and used it as a cathode for RMBs [69]. On the one hand, the introduced abundant oxygen vacancies facilitate the ion transport; on the other hand, a part of Ti^4+^ is reduced to Ti^3+^ accompanied by introducing oxygen vacancies, which reduces the band gap and improves the electronic conductivity of B-TiO_2-*x*_. Compared with TiO_2_ without oxygen vacancies, the kinetic properties of B-TiO_2-*x*_ are greatly improved. In another work, Li’s team reported a Mo-doped VS_4_ cathode for RMBs [70]. The replacement of V^4+^ (0.58 Å) by Mo^4+^ (0.65 Å) expands the interlayer spacing, facilitating the diffusion of Mg^2+^. Moreover, the introduction of Mo forms a Mo-S bond (one Mo bonding with two S), resulting in the initial V-S bond (one V bonding with four S) breaking, thus generating an isolated S, and the isolated S then escapes, inducing sulfur vacancies. The Mo-doping not only facilitates the diffusion of Mg^2+^, but also enhances the electronic conductivity of VS_4_. As a result, the Mo-doped VS_4_ cathode exhibits excellent electrochemical properties. In addition, Mai’s team improved the Mg storage properties of *α*-MoO_3_ through F^−^ substitution doping and H^+^ interstitial doping (denoted as HMoOF) via hydrothermal and metal–acid strategies [68]. The substitutional-doped F^−^ creates Mo vacancies, which unlocks the inert basal plane of *α*-MoO_3_ and accelerates the diffusion of Mg^2+^ along the *b*-axis. H^+^ interstitial doping can, on the one hand, expand the interlayer spacing to lower the migration barrier of Mg^2+^ thus accelerating ion diffusion, and on the other hand, the introduced H^+^ can work as a pillar to stabilize the interlayer structure. Moreover, the dual doping of anions and cations triggers shallow impurity levels (acceptor and donor levels) that reduce the bandgap, thus significantly improving the electrical conductivity of the material. (The conductivity of *α*-MoO_3_ and HMoOF are 2.19 × 10^–7^ and 1.14 × 10^–2^ S cm^−1^, respectively.)

### Increasing Mg^2+^ Storage Sites

The quantity of active sites or Mg storage sites affects the capacity of RMBs. Introducing defects not only enhances the diffusion kinetics of Mg^2+^ but also increases the active sites or Mg storage sites of the host material. With the generation of transition metal cation vacancies, the diffusion channels of Mg^2+^ can be enlarged and Mg storage sites are also increased [71]. The generated cation vacancies can be occupied by Mg^2+^ for reversible insertion and deinsertion. Redox sites can also be increased by doping. New species are introduced into the host material to participate in redox reactions and increase redox sites [63]. The introduction of doping leads to phase transformation and the creation of vacancies, which can possess abundant the active site [72]. In addition, by doping, the interlayer spacing can be expanded, which exposes more active sites [70, 73]. Representatively, an aliovalent-doping anatase TiO_2_ (F–TiO_2_) was reported by Ma and co-workers, in which abundant charge-compensating Ti vacancies (22%) were formed [71]. These vacancies not only facilitate the diffusion of Mg^2+^ activating the Mg storage capacity of anatase TiO_2_ but also act as insertion sites for Mg^2+^, thus enabling the host material to store extra Mg^2+^. Ultimately, the F–TiO_2_ exhibits greatly enhanced reversible capacity and rate performance. Compared to pristine TiO_2_, Ti-deficient TiO_2_ has about six times higher Mg storage capacity, from 25 to 155 mAh g^−1^.

### Strengthening Structural Stability

The stability of the material structure affects the cycling life of the battery. Certain volume changes will be produced by the continuous insertion and deinsertion of Mg^2+^ during cycling, which can affect the structural stability of the material. In addition, the insertion of Mg^2+^ could cause harmful phase transitions of the host material, resulting in irreversible structural damage. Through doping, the Mg^2+^ migration channels can be broadened to reduce the volume changes caused by Mg^2+^ intercalation/deintercalation and improve the structural stability of the host material [74]. The introduction of new species can inhibit harmful phase transitions as well as dissolution. The structural stability of the host material can also be improved by introducing defects through nonstoichiometric synthesis [75, 76]. Particularly, for layered structures, the introduction of new species in the layers can serve as pillars to effectively strengthen the stability of the layer structure [77–79]. For example, Mai’s team developed a bilayer-structured Mg_0.3_V_2_O_5_·1.1H_2_O, with a synergistic effect of Mg^2+^ and lattice water [73]. The pre-inserted Mg^2+^ and lattice water act as pillars, which can stabilize the structure of the material and ensure long-life cycling stability. The Mg_0.3_V_2_O_5_·1.1H_2_O cathode exhibits an ultralong-life cycling performance with capacity retention of 80.0% after 10,000 cycles at 2 A g^−1^.

In summary, proper defects can bring favorable effects to inorganic cathode materials, but inappropriate defects can also result in adverse effects. Although the introduced vacancy defects can improve the diffusion kinetics of Mg^2+^ and increase the Mg storage sites, excessive vacancies will damage the structural stability of the host material. In addition, too many vacancies can also deteriorate the Mg/M (transition metal) anti-site disorder, in which the transition metal ions occupy the insertion sites of Mg^2+^, impeding the diffusion of Mg^2+^, thus leading to capacity decay and structural degradation. Doping by introducing a moderate number of soft anions can effectively reduce the electrostatic interaction between Mg^2+^ and anion lattice, facilitating the diffusion of Mg^2+^. However, an excessive introduction of soft anions will sacrifice the voltage plateau, leading to low-energy density. The structural stability of the host material can be maintained by introducing appropriate inert cations for doping. Since inert cations do not participate in the redox reactions, the excessive introduction of inert cations will reduce the energy density. It should be noted that the realization of these three functions does not necessarily occur simultaneously in a specific case. It is therefore necessary to analyze the effect of defects on Mg^2+^ migration on a case-by-case basis. There is no general empirical formula to balance the positive and negative effects of introducing defects in the RMB field. A combination of experimental and theoretical calculations is still needed for continuous exploration.

## Applications of Defect Engineering to Mg^2+^ Migration in Cathode Materials

The introduction of defects can essentially enhance the diffusion of Mg^2+^ by altering the chemical composition of the host material, improving the electronic conductivity, increasing the Mg storage sites, and enhancing the structural stability of the host material. Then, clarifying exactly what kinds of defects are available and how they are introduced is crucial for the further development of inorganic cathode materials. In this section, based on the previously reported work, an inventory of defect engineering in inorganic cathode materials for RMBs is presented, including the main types of defects and strategies for their introduction.

### Vacancy Defects

Vacancy defect is a form of defect engineering resulting from the absence of ions from lattice sites. The introduced vacancies affect the local structure and charge distribution of the cathode material, facilitate the diffusion of Mg^2+^, and even increase the Mg storage sites. As shown in Fig. 7, vacancy defects can be divided into anionic vacancies and cationic vacancies based on the type of missing ions.

#### Anionic Vacancies

**Fig. 8:**
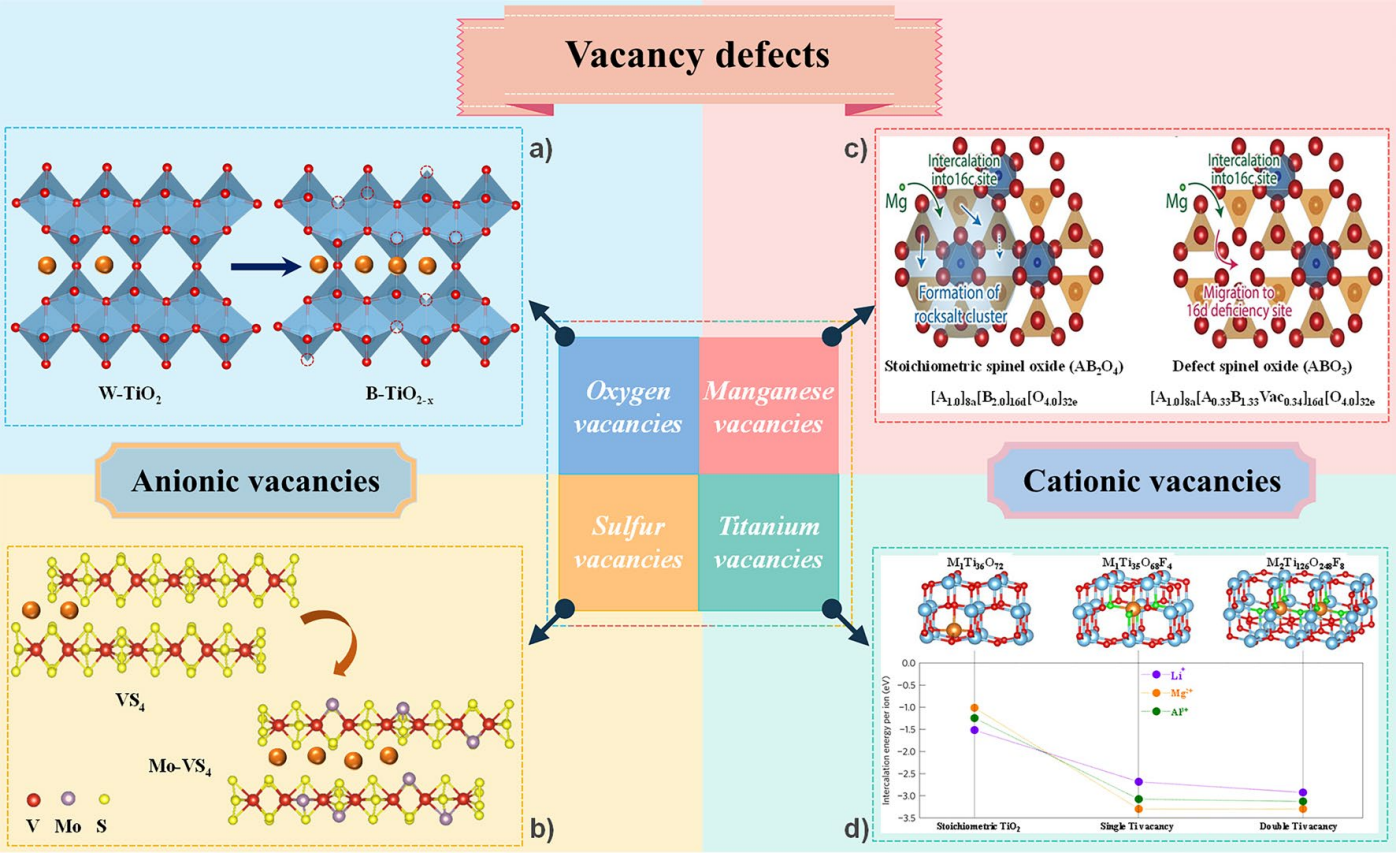
**a** Schematic synthesis process of O vacancies-rich and porous B-TiO_2-*x*_ nanoflakes, **b** Preparation process of Mo-VS_4_/N-TG, and the cycling performance and rate performance. **c** Expected Mg insertion pathways for stoichiometric spinel and defect spinel oxides [75]. Reproduced with permission. Copyright 2021, Wiley–VCH GmbH. **d** Cation-deficient anatase TiO_2_: Titanium vacancies enabling insertion of Mg^2+^ [71]. Reproduced with permission. Copyright 2017, Nature Publishing Group

As discussed previously, Mg^2+^ need to travel through the anion-sharing surface when diffusing in the cathode material, and the strong electrostatic interaction between anion and Mg^2+^ will hinder the migration of Mg^2+^. The introduction of anionic vacancies can reduce the interaction between Mg^2+^ and anionic lattice, thus effectively accelerating the diffusion. For example, Jin et al*.* used an atomic substitution strategy to prepare ultrathin black TiO_2-*x*_ (B–TiO_2-*x*_) nanosheets with rich oxygen vacancies and porosity using ultrathin 2D TiS_2_ nanosheets as precursors (Fig. 8a) [69]. The introduced oxygen-rich vacancies play an important role in the reversible storage of Mg^2+^, both by improving the electrical conductivity (with narrower bandgap) and increasing the number of active sites for Mg^2+^ storage. The DFT calculations indicate that the band gap of B-TiO_2-*x*_ was about 1.79 eV, which is much narrower than that of TiO_2_ without oxygen vacancies (~ 3.30 eV). The kinetics and Mg^2+^ storage capacity of the ultrathin B-TiO_2-*x*_ nanosheets enriched with oxygen vacancies are significantly improved compared with the W–TiO_2_ control sample without oxygen vacancies. Zhao et al. prepared a binder-free and honeycomb V_2_O_5-*x*_ electrode with rich oxygen vacancies on Ti foil (denoted as Ti–V_2_O_5-*x*_) by a facile method of hydrothermal under acidic conditions and calcination in an inert atmosphere [80]. From the GITT tests, the Mg^2+^ diffusion coefficients of Ti–V_2_O_5-*x*_ is in the order of 10^–12^ ~ 10^–14^ cm^2^ s^−1^, which is higher than that of Ti-V_2_O_5_ without oxygen vacancies (10^–13^ ~ 10^–15^ cm^2^ s^−1^). And, the Ti–V_2_O_5-*x*_ electrode has a lower resistance of charge transfer of 206.4 Ω while that of Ti-V_2_O_5_ electrode is 382.6 Ω. Compared with the control sample without oxygen vacancies, the Ti-V_2_O_5-*x*_ electrode exhibits excellent cycling stability and rate performance. It delivers a high initial discharge capacity of 241.3 mAh g^−1^ at 100 mA g^−1^ and maintains a high reversible discharge capacity of 195.4 mAh g^−1^ after 400 cycles. Moreover, it exhibits a discharge capacity of 148.0 mAh g^−1^ at 500 mA g^−1^. Li et al*.* used a facile hydrothermal method, through Mo-doping and nitrogen-doped tubular graphene (N-TG) introduction for decorating VS_4_, to *in*-*situ* synthesize Mo–VS_4_/N-TG with hierarchical structure [81]. Due to the Mo-doping, rich sulfur vacancies were induced to be generated (Fig. 8b). In addition, the highly conductive backbone material N-TG enables the active materials to be dispersed and form tight junctions and enhance electrical conductivity. As the DFT calculation shown the adsorption energy for Mg^2+^ of Mo–VS_4_/N-TG is -6.341 eV which is larger than that of VS_4_ (− 0.506 eV). It suggests that surface active sites have increased and adsorption stability has enhanced with the introducing N-TG and the generating of sulfur vacancies. Moreover, the Mg^2+^ diffusion coefficients of Mo-VS_4_/N-TG range from 1.27 × 10^–13^ to 1.39 × 10^–11^ cm^2^ s^−1^ during the charge state, and from 6.60 × 10^–13^ to 2.63 × 10^–11^ cm^2^ s^−1^ during the discharge state, which is much larger than that of VS_4_/N-TG (from 0.07 to 0.63 × 10^–11^ cm^2^ s^−1^ during the charge state, and from 4.60 × 10^–13^ to 0.73 × 10^–11^ cm^2^ s^−1^ during the discharge state). As a result, Mo-doping and the introduction of N-TG synergistically accelerate the ion diffusion kinetics, ensuring robust structural stability and exposing more Mg^2+^ adsorption active sites.

#### Cationic Vacancies

The introduction of cationic vacancies can reduce the formation of harmful phases (such as the rocksalt phase) that blocks the migration channel, thus keeping the diffusion channel unobstructed. To address the problem that the inserted Mg^2+^ tends to form MgO-like rocksalt clusters in the parent phase leading to poor cyclic stability, Shimokawa et al*.* exploited a defective spinel-type structure to avoid the spinel-to-rocksalt reaction and ensure an unobstructed migration path for Mg^2+^ [75]. In the stoichiometric spinel oxide [A(II)B(III)_2_O_4_], the insertion of Mg^2+^ into the octahedral 16*c* site causes the A(II) cation located at the tetrahedral 8*a* site to become unstable due to the strong electrostatic interactions [82], which leads to the migration of the A(II) cation from tetrahedral 8*a* to the adjacent 16*c* site and the formation of the rocksalt structure (Fig. 8c). The formed rocksalt structure will block the migration path of Mg^2+^ in the spinel-type host structure. However, the defective spinel structure with cation vacancies can solve this problem well, where the vacancies at the 16*d* sites and part of the 8*a* sites can avoid the generation of rocksalt phase to a certain extent and secure the diffusion channel of Mg^2+^ (Fig. 8c). As a result, the defect spinel-type ZnMnO_3_ shows good cyclability.

Besides, the introduction of cationic vacancies can also effectively improve the Mg storage performance of the materials, because it can reduce the electrostatic repulsion between the cationic lattice and Mg^2+^ on the one hand, and the cationic vacancies can act as extra Mg storage sites on the other hand. For example, Ma et al*.* utilized HF etching by introducing Ti vacancies and monovalent doping (F^−^, OH^−^) to make the anatase-structured TiO_2_, which has poor electrochemical activity for multivalent ions, presents reversible Mg storage (Fig. 8d) [71]. Due to kinetic limitations, although Mg^2+^ and Li^+^ exhibit similar insertion energies in stoichiometric TiO_2_ at the dilute limit, pure TiO_2_ displays a poor Mg storage capacity compared to lithium [83]. The use of defect engineering can activate the Mg storage capacity of anatase TiO_2_. As the DFT calculations shown that the intercalation energies of Mg^2+^ in stoichiometric anatase TiO_2_ is −1.02 eV. After introducing Ti vacancies, the Mg^2+^ intercalate more readily at Ti vacancy sites, with intercalation energies of −3.30 eV. The introduction of a large number of titanium vacancies (22%) adds extra Mg^2+^ storage sites, providing a thermodynamically favorable driving force for Mg^2+^ insertion. At the same time, the substitutional doping of F, where F^−^ replaces part of the O^2−^ ion, favors the reduction of the migration barrier of the guest cation. As a result, Ti_0.78_□_0.22_O_1.12_F_0.40_(OH)_0.48_ exhibits a high reversible capacity of about 155 mAh g^−1^, a value about six times higher than that of stoichiometric TiO_2_. The F-doping and high concentration of Ti vacancies remove the kinetic limitation of Mg^2+^ diffusion.

Generally, the introduction of vacancies can reduce the electrostatic interaction between Mg^2+^ and the host lattice (anionic or cationic lattice), lowering the diffusion energy barrier, and thus facilitating the diffusion of Mg^2+^. The introduced vacancies can also act as intercalation sites for Mg^2+^, to increase the Mg storage capacity. Moreover, the free electrons generated by the introduced vacancies can change the valence state of original elements or be able to migrate freely in the lattice, which changes the local electron distribution of the host lattice and improves the electrical conductivity. In particular, by generating suitable cation vacancies, Mg^2+^ can migrate via cation deficiency sites, thus reducing the generation of detrimental phases. Notably, although the introduction of vacancy defects can accelerate ion migration and increase the active sites, the appropriate concentration of vacancies needs to be controlled because too many vacancies can adversely affect the structural stability of the material. Owing to the limited research, it is still unclear the mechanism of vacancy defects in some cathode materials for RMBs and further investigations are needed. It is also worth exploring how to rationally and precisely create vacancies.

### Doping Defects

In addition to vacancy defects, doping defect is a very valuable way to improve the diffusion kinetics of Mg^2+^. The introduction of doping changes the local electron distribution, exposes more active sites, increases conductivity, improves reaction kinetics, and enhances structural stability. The doping defect is divided into substitutional doping and interlayer doping, and the form of doping can be ionic, molecular, or even protonic doping.

#### Substitutional Doping

Substitution doping refers to the introduction of heterogeneous ions at specific lattice sites to replace original ions. The introduction of cations for doping is called cationic doping. For example, Kim et al*.* used a hydrothermal method for Cr doping of lithium titanate (n-Cr-LTO) [84]. From the electrochemical impedance spectroscopy (EIS) measurements, the charge transfer resistance of n-Cr-LTO is 13 Ω, which is much smaller than that of n-LTO (75 Ω), verifying the beneficial effect of enhanced electrical conductivity. In addition, the migration barrier in n-Cr-LTO is 0.30 eV, which is lower than that of undoped LTO (0.70 eV), indicating that Mg^2+^ migration can be more facile by introducing Cr doping in LTO. The aliovalent doping of Cr induces beneficial structural disorder in LTO, which lowers the migration barrier of Mg^2+^ and thus promote rapid Mg^2+^ diffusion. This work improves the reaction kinetics of the Mg storage in LTO and offers a new possibility for the use of conventional intercalation-type materials in RMBs. Moreover, Zhuo et al*.* synthesized a hierarchical nanocapsule of hydrogen-substituted graphdiyne (HsGDY) nanotube encapsulated with Cu-doped MoS_2_ nanopetals and implanted buffer zones by a solvothermal method [85]. The MoS_2_ nanometals introduced into Cu expose abundant active sites, and the extended π-conjugated structure of HsGDY provides more efficient electron and ion transfer channels. The Cu–MoS_2_@HsGDY capsules have a specific surface area and pore volume of 168 m^2^ g^−1^ and 0.627 cm^3^ g^−1^, respectively, and exist two typical mesopores (12 and 34 nm), which provide considerable diffusion channels and contact area for the electrolyte. Besides, the Cu–MoS_2_@HsGDY electrode has a charge transfer resistance of 9.83 Ω, which much lower than that of MoS_2_ (111.70 Ω), suggesting the facilitated electron transfer. Therefore, the material exhibits good electrochemical performance. The Cu-doped MoS_2_ nanopetals demonstrate a reversible capacity of 148.5 mAh g^−1^, which is six times higher than that of MoS_2_ (23.5 mAh g^−1^). In short, the introduction of different ions for doping has different effects and the corresponding ions need to be selected for doping according to specific needs. However, the concentration of doping needs to be controlled to achieve the stated purpose without sacrificing energy density.

**Fig. 9:**
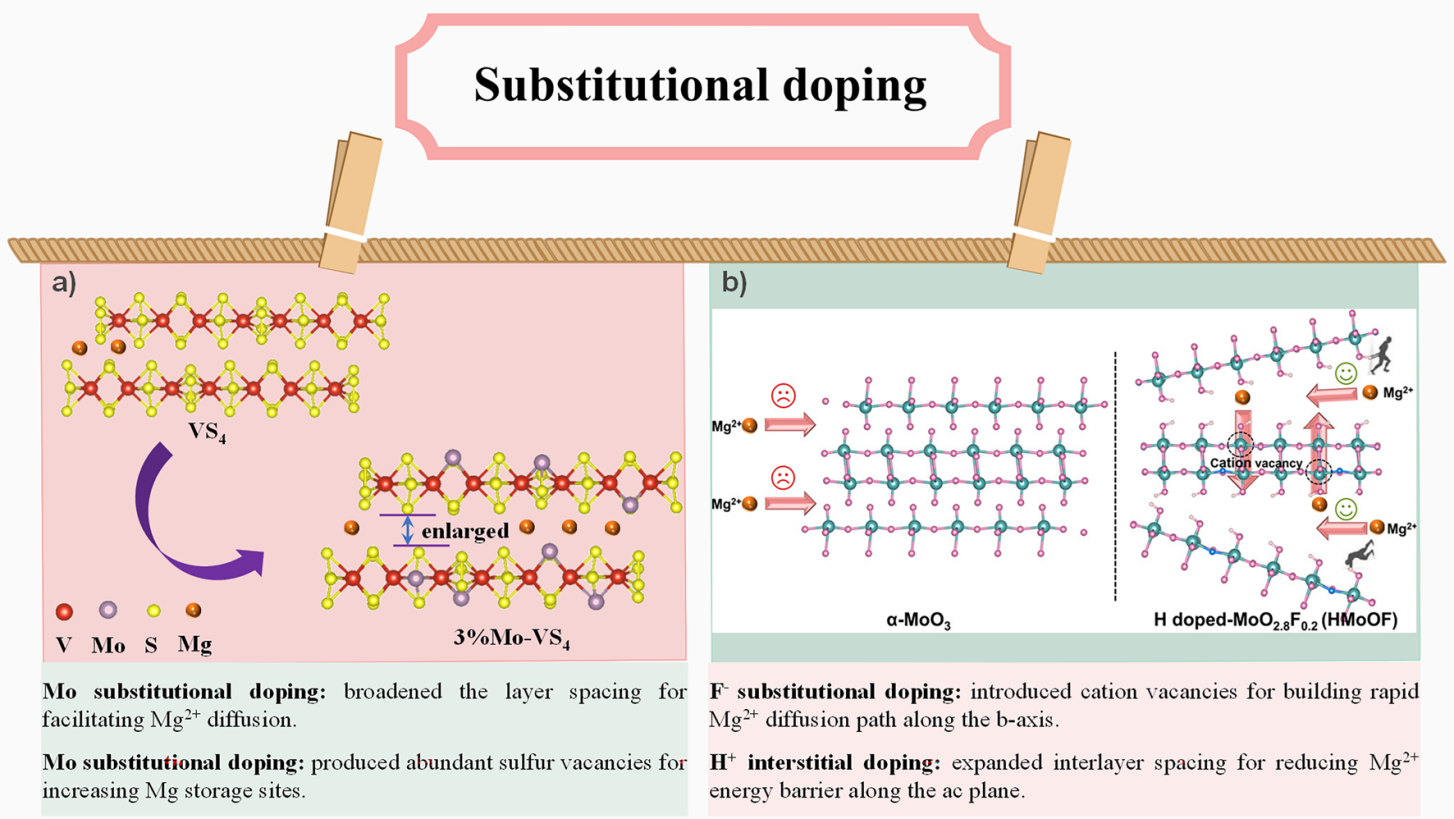
**a** Microstructure regulation by Mo-doping on 3% Mo-VS_4_, **b** F^−^ substitutional and H^+^ interstitial doping for α-MoO_3_ (HMoOF) [68]. Reproduced with permission. Copyright 2022, American Chemical Society

In addition, the introduction of cations for cationic doping may lead to the creation of anionic vacancies. Representatively, Li et al*.* synthesized Mo-doped VS_4_ by a simple one-step hydrothermal method and found that Mo-doping broadened the layer spacing and produced abundant sulfur vacancies (Fig. 9a) [70]. On the one hand, since the ionic radius of Mo^4+^ is slightly larger than that of V^4+^, Mo-doping is beneficial to expanding the interlayer spacing, which facilitates Mg^2+^ diffusion; on the other hand, the abundant sulfur vacancies can be served as active sites for Mg^2+^ adsorption and desorption, which also accelerates the ion diffusion. The Mg^2+^ diffusion coefficients calculated from GITT curves indicate that rich sulfur vacancies and expanded interlayer spacing by Mo-doping have played a major role in facilitates the diffusion of Mg^2+^. The Mg^2+^ diffusion coefficients of 3% Mo–VS4 range from 1.10 × 10^–12^ to 15.70 × 10^–12^ cm^2^ s^−1^ during the charge state, and from 1.82 × 10^–12^ to 5.75 × 10^–12^ cm^2^ s^−1^ during the discharge state, which is much larger than that of VS_4_ (from 0.69 × 10^–12^ to 1.84 × 10^–12^ cm^2^ s^−1^ during the charge state, and from 0.63 × 10^–12^ to 2.87 × 10^–12^ cm^2^ s^−1^ during the discharge state). Finally, the Mo-doped VS_4_ obtains a high reversible specific capacity about 120 mAh g^−1^ after 350 cycles at 50 mAg^−1^, while VS_4_ exhibits fast capacity decay (only 48.8 mAh g^−1^ after 120 cycles) without Mo-doping. Besides, the Mo-doped VS_4_ exhibits better rate performance even at 500 mAg^−1^.

Similarly, anionic doping can also lead to the generation of cationic vacancies. For example, Mai’s team prepared F^−^ and H^+^-doped MoO_3_ materials (denoted as HMoOF) through hydrothermal as well as metal–acid strategies [68]. F^−^substitutional doping leads to the generation of Mo vacancies, which unlocks the initially inert basal plane of MoO_3_, accelerating Mg^2+^ migration along the b-axis. Moreover, the interstitial-doped of H^+^ enlarges the interlayer spacing and serves as a pillar to stabilize the interlayer structure, which reduces Mg^2+^ diffusion energy barriers along the ac plane, thus realizing a three-dimensional channel for rapid Mg^2+^ diffusion (Fig. 9b) [68]. As a result, the HMoOF electrode exhibits an outstanding reversibility, displaying a capacity of 276.3 mAh g^−1^ after 100 cycles at 0.1 A g^−1^, which is much higher than those of α-MoO_3_ and MoOF (only with F^−^ doping) electrode. The calculated conductivity of α-MoO_3_, MoOF, and HMoOF are 2.19 × 10^–7^, 8.8 × 10^–3^, and 1.14 × 10^–2^ S cm^−1^, respectively. The corresponding band gap of the HMoOF is 1.38 eV, which is much lower than that of α-MoO_3_ (3.01 eV) and MoOF (1.74 eV). And, the Mg^2+^ diffusion coefficient of the HMOF electrode is 2.32 × 10^–11^ to 2.89 × 10^–13^ cm^2^ s^−1^, which is much higher than that of both pure MoO_3_ electrode and MoOF electrode. Therefore, compared with MoO_3_ and MoOF, the HMOF electrode exhibits better rate performance, even demonstrating a specific capacity of 137.4 mAh g^−1^ at 2 A g^−1^.

To sum up, substitutional doping enhances the electronic conductivity of the material by introducing new ions into the host lattice, changing the original electronic distribution and elemental valence state. For insertion-type inorganic materials, the transition metal cation generally acts as the redox center, which determines the redox potential and the number of transferred electrons (*i.e.*, the number of stored Mg ions). Therefore, by introducing suitable active metal cations to participate in the redox reaction, the redox potential can be regulated as well as exhibit good Mg storage properties. The introduction of inert metal cations, although not contributing to the redox reaction, maintains structural stability. Excessive introduction of inert cations reduces the energy density. Besides, by introducing softer anions to replace part of the original anions, the interaction between Mg^2+^ and anion lattice is reduced to some extent, thus improving the sluggish diffusion. Although the diffusion of Mg^2+^ is improved by introducing softer anions for substitution doping, this comes at the cost of lowering the voltages, thereby reducing the energy densities of cathode materials. It should be considered that the concentration of the introduced ions needs to be moderate as the excessive introduction of ions can generate new phases and not achieve the desired effect. Theoretically, the synergistic use of cationic and anionic doping could combine their advantages to simultaneously improve redox potential, Mg^2+^ diffusion, and structural stability. However, there are few attempts in this area, which may be attributed to the complex preparation process it involves.

#### Interlayer Doping

**Fig. 10:**
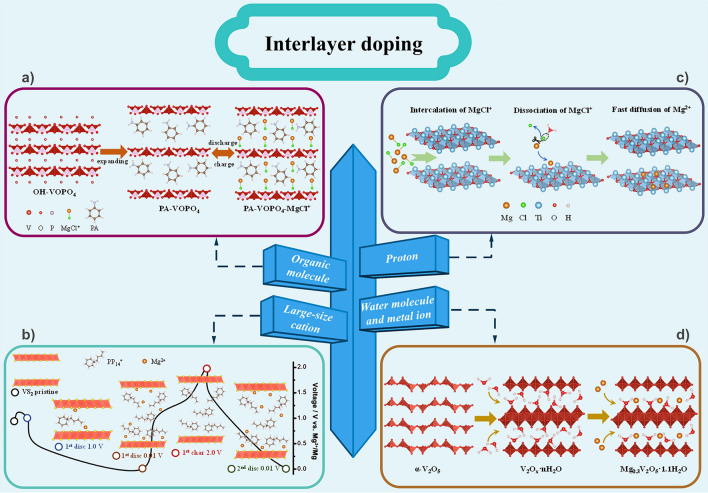
**a** Schematic illustration of the experimental approach and proposed mechanism of PA-VOPO_4_ nanosheets as Mg storage materials. **b** Schematic of structural evolution of VS_2_ during discharge/charge process. **c** Schematic illustration of the mechanism of genuine Mg^2+^ storage through protons in the designed oxide electrode. **d** Schematic diagrams of the formation of the bilayer-structured Mg_0.3_V_2_O_5_·1.1H_2_O

For layered structures, organic molecules, large-sized cations, water molecules, protons or metal ions can be pre-inserted into the layers, which improve the Mg storage properties of the material. The introduction of small organic molecules in the layers increases the layer spacing. For example, Yao’s team expanded the interlayer distance of MoS_2_ from 0.615 to 1.45 nm by inserting poly(ethylene oxide) (PEO) into the MoS_2_ interlayers [86]. The expansion increases the diffusivity of Mg^2+^ by two orders of magnitude (from 10^–13^ to 10^–11^ cm^2^ s^−1^), and raises the specific capacity of the barely active MoS_2_ over 3.4 times (from 22 to 75 mAh g^−1^). In addition, Mai et al*.* realized pre-intercalation of phenylamine (PA) into VOPO_4_ layers via a facile ultrasonicated exfoliation and self-assembly route, resulting in the increase of the layer spacing from 0.74 to 1.42 nm (Fig. 10a) [87]. The extended layer spacing accommodates the insertion of MgCl^+^, which essentially reduces the polarity barrier, alleviates diffusion kinetics, as well as exposes extra Mg storage sites. As the DFT calculations shown, the MgCl^+^ diffusion exhibits an energy barrier of 0.42 eV, much lower than that of Mg^2+^ (1.20 eV). And, the diffusion coefficient of MgCl^+^ is 1.5 × 10^13^ times that of Mg^2+^. As a result, the PA-VOPO_4_ electrode exhibits a high reversible capacity of 310 mAh g^−1^, high rate performance, and long cycle life (192 mAh g^−1^ at 100 mA g^−1^ even after 500 cycles). In another work, Wei et al*.* developed an APCP_14_Cl/THF electrolyte for RMBs (Mg metal as anode and VS_2_ nanosheets as the cathode), using 1-buty-1-methylpiperidinium chloride (PP_14_Cl) as an additive [88]. As indicated in Fig. 10b, during the first discharge, the large-sized PP_14_^+^ is embedded in the VS_2_ nanosheets and permanently resides in the material. Therefore, the layer spacing of VS_2_ is broadened, which increases the diffusion coefficient of Mg^2+^ by three orders of magnitude (from 10^–13^ ~ 10^–15^ to 10^–10^ ~ 10^–12^ cm^2^ s^−1^). Furthermore, the embedded positively charged PP_14_^+^ attracts the Cl^−^ of MgCl^+^, thus reducing the Mg^2+^ desolvation energy from 3.0 to 0.67 eV. As a result, it exhibits a capacity of 348 mAh g^−1^ at 20 mA g^−1^ and excellent rate capability of 214 mAh g^−1^ at 2.0 A g^−1^. It should be noted that the widening of the layer spacing by introducing small organic molecules needs to be moderate. Excessive expansion of the layers is undesirable because it sacrifices volumetric energy density.

The water molecule can easily solvate Mg^2+^ due to its strong polar dipole, thus forming hydrated Mg(H_2_O)_6_^2+^, which shields the strong polarization of Mg^2+^ and also reduces its electrostatic interaction with the host ion, ultimately improving the diffusion kinetics. Therefore, the introduction of water molecules into the layers lubricates the solid-state diffusion of Mg^2+^ and reduces the migration energy barrier of Mg^2+^ due to the charge shielding effect. Recently, Wang et al*.* fabricated a water-activated layered structure of VOPO_4_ as a novel cathode material using a simple hydrothermal method and investigated the role of water in the electrode or organic electrolyte on the thermodynamics and kinetics of Mg^2+^ insertion/extraction in the cathode [89]. The results show that the structural water in the layers expands the interlayer distance (from 4.17 to 7.41 Å) and lubricates the diffusion of Mg^2+^, thus reducing the electrostatic interaction. The diffusion barriers of Mg^2+^ in VOPO_4_·H_2_O are 0.48 eV, lower than that of VOPO_4_ (1.56 eV), and the diffusion coefficient of Mg^2+^ in VOPO_4_·H_2_O is 1.2 × 10^18^ times higher than that of VOPO_4_. In a dry electrolyte (without water molecules), the Mg^2+^ solvates with propylene carbonate (PC) molecules to form [Mg(PC)_6_]^2+^, while in a wet electrolyte (containing water molecules), Mg^2+^ prefers to solvate with water rather than PC molecules. The large solvation energy (2.27 eV) of [Mg(PC)_6_]^2+^ makes it difficult for Mg^2+^ to desolvate and insert into the cathode material. In contrast, water molecules co-intercalate with partially desolvated Mg^2+^, which facilitates the intercalation of Mg^2+^. As a result, the water molecule activates the Mg storage capacity of the cathode material. Dialectically, although the introduction of water molecules lowers the migration barrier of Mg^2+^ and accelerates its diffusion, its compatibility with Mg metal anode and organic electrolyte needs to be considered. In addition, it is not clear whether the introduced water molecules will be participating in the electrochemical reactions.

In all phenyl complex (APC) electrolyte, the cathode material usually stores MgCl^+^ instead of directly storing Mg^2+^, which will continuously consume the electrolyte and lead to a lower energy density, although MgCl^+^ ions can reduce their interaction with the host lattice compared to Mg^2+^ [32]. To achieve genuine Mg^2+^ storage, the Cl^−^ in MgCl^+^ needs to be removed. Thus, Geng et al*.* introduced a proton-assisted method to dissociate the Mg–Cl bond to enable genuine Mg^2+^ insertion into the oxide host lattice by placing protons on negatively charged metal-deficient oxide sheets (Fig. 10c) [90]. The protons are adsorbed as hydrated hydrogen ions or hydroxyl groups on the oxygen atoms at the vertices of the TiO_6_ octahedra. Theoretical calculations show that in the presence of H_3_O^+^ ions, the cleavage energy of the Mg–Cl bond significantly decreases from 2.82 to 0.86 eV, and the bond distance increases from 0.22 to 0.24 nm, which indicates that the presence of protons facilitates the dissociation of Mg–Cl bond.

Metal ions can be pre-inserted into the layers to act as pillars, where the pre-inserted metal ions coordinate with the host anion, thus modifying the interlayer spacing. Moreover, the electrostatic interaction between the metal ion and the host anion plays a role in stabilizing the layered structure. In addition, the electrostatic repulsion between metal ions and Mg^2+^ facilitates the diffusion of Mg^2+^. It is not negligible that metal ions are smaller or even lighter than organic molecules, which does not reduce the energy density of the material. Mai et al*.* developed a bilayer-structured vanadium oxide (Mg_0.3_V_2_O_5_·1.1H_2_O) with a synergistic effect of Mg^2+^ and lattice water as a cathode material for RMBs via a simple hydrothermal method (Fig. 10d) [73]. The lattice water not only widens the lattice spacing of the cathode material allowing the insertion and extraction of Mg^2+^ but also shields the charge of Mg^2+^, leading to rapid diffusion. The pre-inserted Mg^2+^ act as pillars to stabilize the layer structure and facilitate electronic conductivity, thus ensuring cycle stability.

In practical applications, defects are introduced to achieve specific goals, such as, improving the ion diffusion coefficient, elevating the intrinsic electronic conductivity, and bolstering the structural stability of the host material. One type of defect can be introduced, such as vacancy defects (anionic vacancies or cationic vacancies), and doping defects (substitutional doping, or interlayer doping like interlayer molecular doping and ionic doping). Of course, multiple types of defects can be introduced to work together, for example, the simultaneous introduction of vacancy defects and doping defects, and dual doping of anions and cations. Table 1 shows the defect engineering and the enhanced electrochemical performance compared with the pristine materials.

**Table 1:** Comparison of electrochemical performance between some cathode with defect engineering and the pristine materials for RMBs

Cathode	Developed strategies	Fabrication methods	Electrolyte	Rate performance (mAh g^−1^/mA g^−1^)	Cycling performance (mAh g^−1^/number/mA g^−1^)	References
W–TiO_2_	–	–	0.4 M APC/THF	~ 48/300	~ 35/400/300	[69]
B–TiO_2-*x*_	Oxygen vacancy	Atomic substitution	0.4 M APC/THF	106/300	77/400/300	
TiO_2_	–	–	0.2 M APC/THF	–	~ 25/3/20	[71]
Ti_0.78_□_0.22_O_1.12_F_0.40_(OH)_0.48_	Ti vacancy and F-doping	Acid etching	0.2 M APC/THF	77/300	100/200/150; 65/500/300	
(Ti_1.74_O_4_)^1.04−^(C_6_H_16_N)^+^_0.36_(H^+^)_0.68_·1.37H_2_O	proton interstitial doping	Proton exchange	0.4 M APC/THF	100/5000	~ 163/2000/500	[90]
VS_4_	–	–	0.4 M APC/THF	20.1/500	74/185/50	[81]
Mo-VS_4_/N-TG	Mo-doping/S vacancy	Hydrothermal reaction	0.4 M APC/THF	76.6/500	140/600/50	
MoO_3_	–	–	0.3 M Mg(TFSI)_2_/acetonitrile	~ 52.0/2000	~ 30.3/100/100	[68]
HMoOF	F^−^ substitutional and H^+^ interstitial doping	Acid treatment	0.3 M Mg(TFSI)_2_/acetonitrile	137.4/2000	~ 142.5/800/1000	
V_2_O_5_·nH_2_O	Pre-intercalated lattice water	Hydrothermal reaction	0.3 M Mg(TFSI)_2_/acetonitrile	~ 34/2000	~ 34/200/100	[73]
Mg_0.3_V_2_O_5_	Pre-intercalated Mg^2+^	Hydrothermal reaction	0.3 M Mg(TFSI)_2_/acetonitrile	~ 24/2000	~ 11/200/100	
Mg_0.3_V_2_O_5_·1.1H_2_O	Pre-intercalated Mg^2+^ and lattice water	Hydrothermal reaction	0.3 M Mg(TFSI)_2_/acetonitrile	85/2000; 50/4000	~ 174/500/100; ~ 118/10000/1000; ~ 87/10000/2000	
Ti–V_2_O_5_	–	–	0.5 M Mg(ClO_4_)_2_/acetonitrile	57.2/500	97.9/400/100	[80]
Ti–V_2_O_5-*x*_	Oxygen vacancy	Hydrothermal reaction	0.5 M Mg(ClO_4_)_2_/acetonitrile	148.0/500	195.4/400/100	
ZnMn_2_O_4_	–	–	0.5 M LiTFSA/DEME-TFSA	–	~ 82/34/10	[75]
ZnMnO_3_	Cationic vacancy	Nonstoichiometric synthesis	0.5 M LiTFSA/DEME-TFSA	~ 45/200	~ 96/120/10	
MoS_2_	–	–	0.5 M MgCl_2_-AlCl_3_/DME	~ 10/1000	~ 23.5/200/50	[85]
Cu–MoS_2_@HsGDY	Cu doping	Solvothermal reaction	0.5 M MgCl_2_-AlCl_3_/DME	91/1000	~ 148.5/200/50; 100/300/200; 85.5/300/500	
Bulk-type Li_4_Ti_5_O_12_	–	–	1.0 M THFPB-0.05 M MgF_2_/DME	~ 6/175	~ 16/156/87.5	[84]
Nanosized Li_4_Ti_5_O_12_	–	–	1.0 M THFPB-0.05 M MgF_2_/DME	47/175	~ 53/170/87.5	
Li_3_Ti_3_Cr_3_O_12_	Cr doping	Hydrothermal reaction	1.0 M THFPB-0.05 M MgF_2_/DME	65/173	~ 74/170/86.5	
Com–MoS_2_	–	–	0.25 M APC/THF	~ 4/500	–	[86]
Peo_2_–MoS_2_	PEO interlayer doping	Chemical delamination-reassembly method	0.25 M APC/THF	~ 20/500	~ 69/30/5	
OH-VOPO_4_	–	–	0.25 M APC/THF	~ 42/2000	~ 122/130/100	[87]
PA-VOPO_4_	PA interlayer doping	Self-assembly method	0.25 M APC/THF	109/2000	192/500/100	
VOPO_4_·2H_2_O	Water interlayer doping	Hydrothermal reaction	0.1 M Mg(ClO_4_)_2_·6H_2_O/PC	–	~ 94/50/5	[89]

**Fig. 11:**
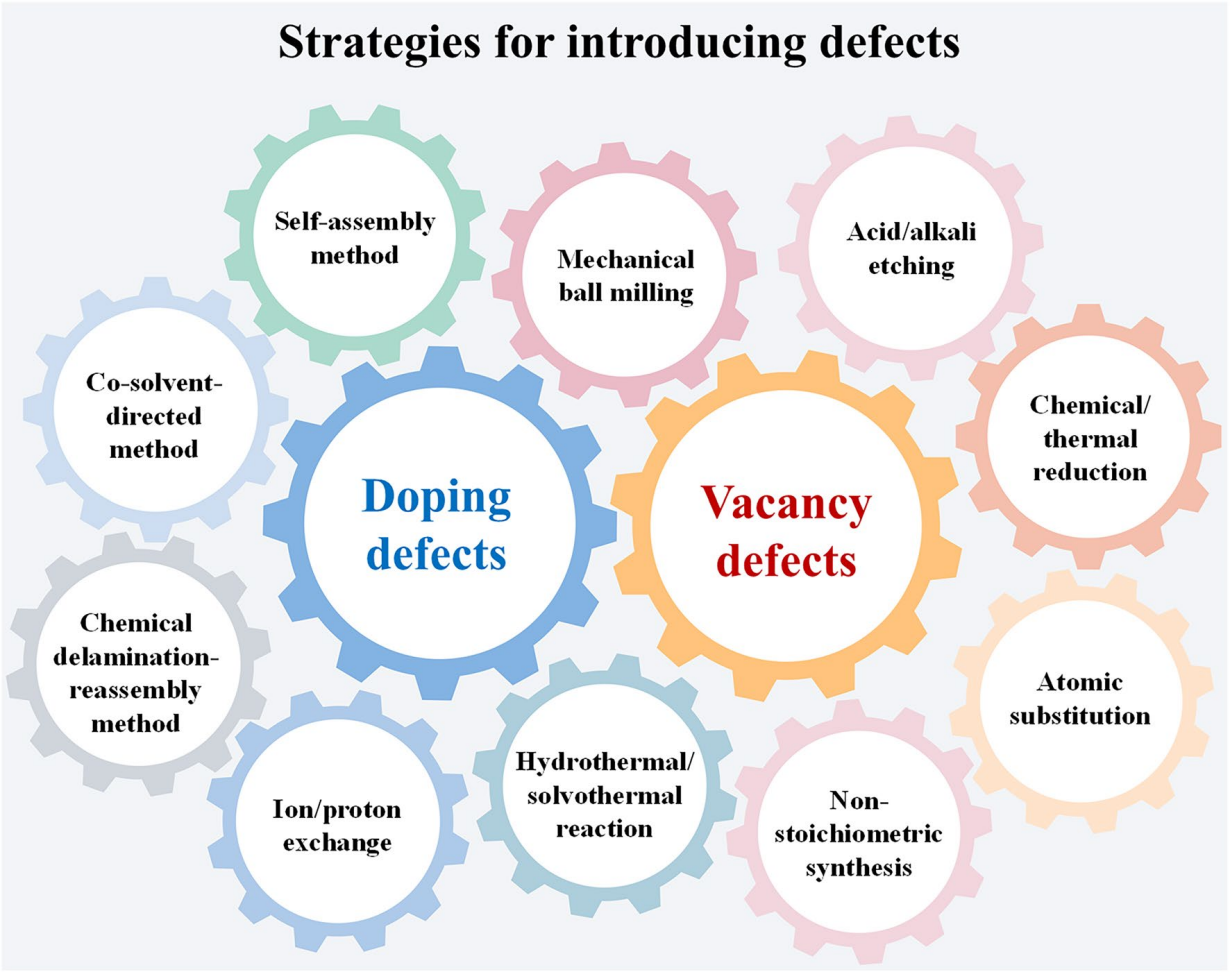
Strategies for introducing defects into inorganic cathode materials

The introduction of defects in the material increases the active site, lowers the migration energy barrier, improves electrical conductivity, and ensures structural stability. From the previous application cases of defect engineering in RMBs, the methods of introducing defects can be broadly classified into physical and chemical strategies (Fig. 11). The physical strategies do not require the introduction of chemical reagents and are relatively simple to operate with the assistance of specific equipment. For example, mechanical ball milling is a common physical method by which the introduction of vacancy defects can be performed. The chemical strategies can introduce not only vacancy defects but also doping defects. For instance, vacancy defects can be introduced by strong acid/alkali etching. And, vacancy defects can also be introduced by chemical reduction and thermal reduction. Besides, nonstoichiometric synthesis is also an effective way to generate vacancy defects. Hydrothermal/solvothermal reactions can be used to introduce both vacancy defects and doping defects. Doping defects can be introduced by co-solvent-directed method as well. Particularly, interlayer molecular doping is performed by the self-assembly method, while ions can be introduced in layers via the ion/proton exchange.

## Summary and Outlook

**Fig. 12:**
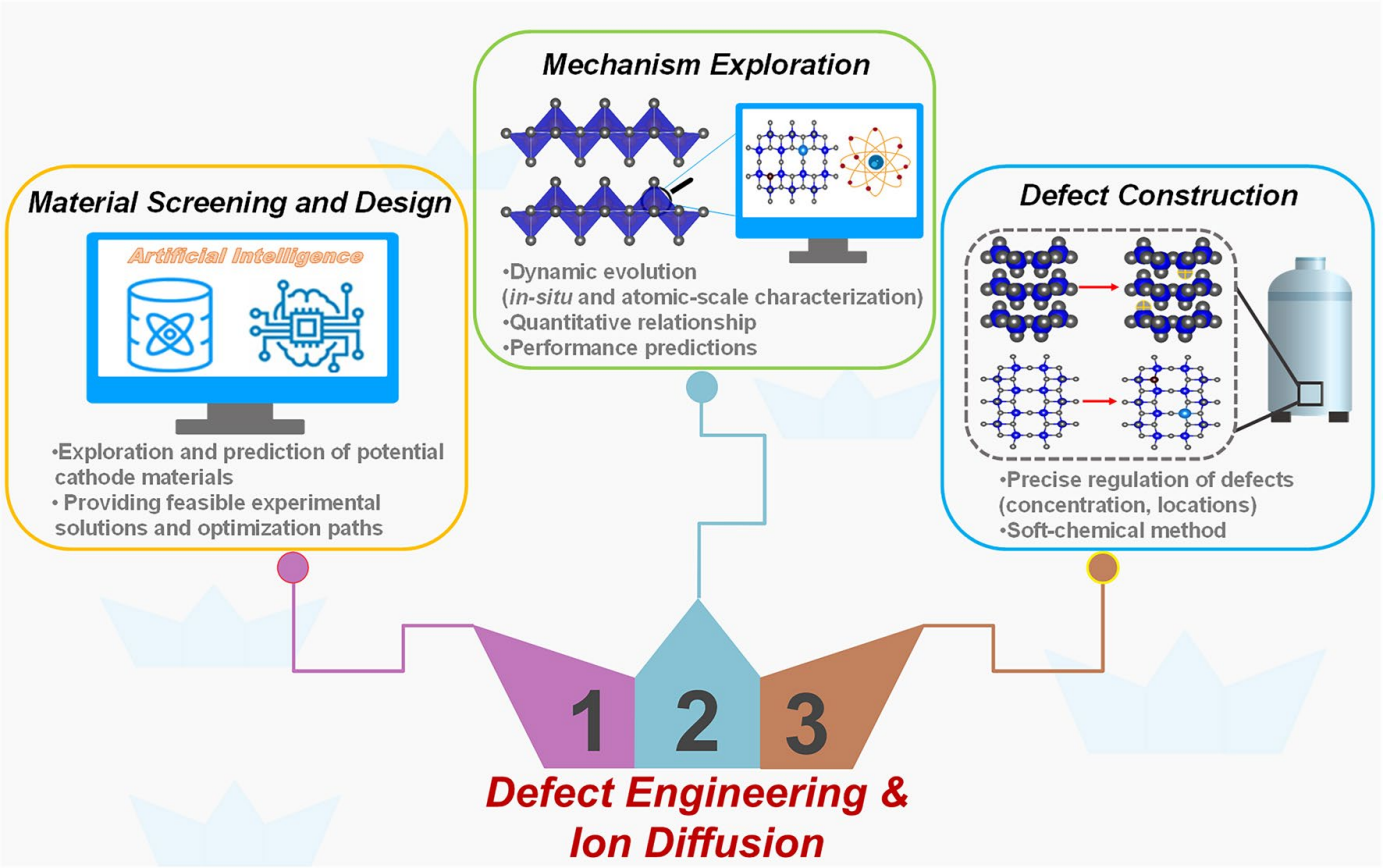
Future perspectives for defect engineering in cathode materials for RMBs

Introducing defects is an effective strategy to improve the slow diffusion kinetics of Mg^2+^ in inorganic cathode materials, increase the Mg storage sites, and strengthen the structural stability of the materials, thus realizing high-performance electrodes for RMBs. This review aims to begin by highlighting the fundamental scientific understanding of the intrinsic mechanism of Mg^2+^ migration and the corresponding affecting factors, and then emphasize the positive effects of defects on the electrochemical performance of cathode materials for RMBs. Moreover, the typical types of defects and various strategies for introducing defects are summarized. Obviously, the introduction of suitable defects can improve the ion diffusion and electron conductivity, increase the active sites, and enhance the structural stability, thus substantially upgrading the Mg storage capacity, rate performance, and long cycling life of the electrode. Despite the advantages of defect engineering have been demonstrated in many applications of cathode materials for RMBs, the understanding of the positive effect of defects on Mg^2+^ diffusion still remains at a relatively superficial level. Below are some development directions that we believe can broaden the scope and depth of defect research (Fig. 12).

### Material Screening and Design Empowered by Artificial Intelligence

Compared to other battery systems, the development of rechargeable magnesium battery systems started late, and there are not many types of high-voltage cathodes. The exploitation of cathode materials through experimental trial-and-error method is very costly. Therefore, the process of high-voltage and high-specific capacity cathode materials screening can be speed up with the aid of high-throughput calculations or even machine learning (ML). High-throughput calculations and machine learning, as core components of artificial intelligence (AI), can discern patterns from high-dimensional data, thereby providing more reliable, reproducible, and accurate results. On the one hand, theoretical calculations can be utilized to explore whether the known cathode materials with excellent performance in other battery systems are also suitable for RMBs; on the other hand, more efficient and accurate predictions of potential cathode materials can be made, providing new avenues for RMB advancement.

Meanwhile, in response to the high cost of the traditional experimental trial-and-error method and the limitations of understanding the material properties, the effects of vacancy concentration, doping element type and doping ratio on the Mg storage performance of high-voltage insertion-type oxide cathode can be systematically investigated with the help of high-throughput calculations. Moreover, the correlation between Mg^2+^ transport behavior and lattice structure, chemical components, and defect configuration can be evaluated, which can provide feasible experimental solutions and optimization paths, as well as deepen the understanding of cathode material design and development.

### Mechanism Exploration Enhanced by Advanced Characterization

It is necessary to further in-depth probe whether and how the local defects in material undergo structural evolution during battery operation, and how these local changes affect the overall performance. Furthermore, the quantitative relationship between introduced defects and electrochemical properties needs to be investigated. Quantifying the concentration effect and the generating sites of the introduced defects on electrochemical properties will facilitate to predict the material’s electrochemical performance and the subsequent precise optimization. Therefore, it is essential to utilize advanced characterization techniques, such as *in situ* characterization and atomic-scale characterization techniques, to probe the local structure as well as the dynamic evolution of the material during operation, to establish the connection between structural units and defect chemistry. In addition, the link between defect engineering and electrochemical performance needs to be established by combining structural characterization data with electrochemical performance analysis.

### Defect Construction with Controlled Enhancement by Soft-Chemical Method

The concentration of defects needs to be controlled in the effective interval as not all generated defects are favorable. Therefore, a scheme that can precisely regulate the concentration and generation locations of defects is required. The author believes that the process of introducing doping defects is more suitable for large-scale production, and the concentration of doping defects is easier to control. Soft-chemical method is very promising for introducing doping defects from a practical standpoint. The equipment required for soft-chemical synthesis is quite simple, the reaction process is easier to control, and the preparation cost is relatively low, making it an environmentally friendly, economical, and efficient means of production. Doping defects such as metal cation/nonmetal cation, anion, small molecule, etc., can be introduced controllably by soft-chemical techniques like sol–gel, coprecipitation, ion exchange, hydrothermal/solvothermal, and acid–base treatment. In addition, a gradient doping can be realized through fine tuning. Doping defects have been widely applied in various energy storage and conversion fields. Regrettably, there are relatively few relevant reports in the field of RMBs, and vast attempts are still needed.

Apart from the slow diffusion in the bulk of the cathode material, the sluggish migration of Mg^2+^ at the electrode–electrolyte interface is also a pressing problem. The migration of Mg^2+^ at the interface needs to overcome the desolvation barrier and undergo a slow desolvation process. Storage of complex ions with larger sizes requires larger diffusion channels to accommodate them and reduces energy density. This issue can be addressed from the electrolyte perspective, such as developing electrolytes that can be properly coupled to facilitate Mg^2+^ desolvation or adding suitable additives to form an effective cathode-electrolyte interphase (CEI) to aid Mg^2+^ desolvation. Of course, it can also be solved from the aspect of cathode materials, such as artificially constructing CEI, or designing suitable surface coating. Currently, few systematic studies on this issue have been reported. It is a direction that needs to be continuously explored in the future.
